# Comparison of work-related musculoskeletal symptoms between male cameramen and male office workers

**DOI:** 10.1186/s40557-018-0243-y

**Published:** 2018-05-02

**Authors:** Han-Seur Jeong, Byung-Seong Suh, Soo-Geun Kim, Won-Sool Kim, Won-Cheol Lee, Kyung-Hun Son, Min-Woo Nam

**Affiliations:** Department of Occupational and Environmental Medicine, Kangbuk Samsung Hospital, Sungkyunkwan University School of Medicine, 29 Saemunan-ro, Jongno-gu, Seoul, 03181 South Korea

**Keywords:** Work-related musculoskeletal disorder, Cameramen, Office worker, Ergonomics, Work environment

## Abstract

**Background:**

Previous studies have classified cameramen’s job as physiologically heavy work and identified the risk factors of work-related musculoskeletal disorders (WRMDs) in cameramen. However, those studies limited their research subjects to cameramen. In this study, we compared the frequency and severity of WRMDs between cameramen and office workers.

**Methods:**

A total of 293 subjects working in four broadcasting companies in Korea were recruited. A questionnaire survey was conducted for a month, starting in October 2016. The subjects were divided into cameramen and office workers according to their occupation. We compared the frequency and severity of WRMDs and ergonomic risk assessment results between the two groups.

**Results:**

The high-risk WRMD group had a higher proportion of cameramen than office workers. Moreover, the high ergonomic risk group also had a higher proportion of cameramen than office workers for WRMDs in the upper extremities and waist+lower extremities. In the multivariable-adjusted model comparing cameramen and office workers, the odds ratio (OR) with 95% confidence interval (95% CI) for high-risk WRMDs was 3.50 (95% CI: 1.92–7.72) for the upper extremities and 3.18 (95% CI: 1.62–6.21) for the waist and the lower extremities. The ORs by body parts were 3.11 (95% CI: 1.28–7.57) for the neck, 3.90 (95% CI: 1.79–8.47) for the shoulders, and 4.23 (95% CI: 1.04–17.18) for the legs and feet.

**Conclusions:**

Our study suggests that cameramen are at high risk of WRMDs. Workplace improvements and management of the neck, shoulders, and lower extremities, which are susceptible to WRMDs, are necessary to prevent musculoskeletal disorders among cameramen.

## Background

Work-related musculoskeletal disorders (WRMDs) refer to muscle, bone, joint, ligament, tendon, or nerve pain that is induced or aggravated by work [1]. Pain caused by WRMDs affects physical health, leading to reduced functional capacity and vitality. Moreover, it also affects mental health, resulting in lower satisfaction with interpersonal relationships and home life and cause additional cost burdens, ultimately reducing an individual’s quality of life [2–4]. WRMDs are known to be one of the main causes of work absence [5, 6]. Buckle and Devereux [7] estimated that 45% of all absences associated with occupational diseases were due to WRMDs. WRMDs are associated with reduced productivity in the society [8, 9]. However, although musculoskeletal disorders can cause problems at the personal and socioeconomic levels, they are significantly underreported [10].

Biomechanical exposure, which is associated with WRMDs, includes static posture; repeated pushing, pulling, lifting, and neck bending [11]; highly repetitive work; and forceful exertion in work [12]. In addition, workplace factors (e.g., time pressure, poor work/rest schedule, and low job control), physical factors (e.g., old age and elevated body mass index), and psychosocial stress have also been reported as risk factors of WRMDs [13]. A study has reported that these ergonomic risk factors are present in workplaces for cameramen and that they affect the incidence of WRMDs among cameramen [14]. Another study has reported that because cameramen perform physically demanding tasks and thus experience high levels of physiological stress, their occupation must be classified as heavy work [15].The environment for making TV programs in Korea lacks production costs and staff, giving cameramen long working hours and short break times. This increases the risk of work-related musculoskeletal disorders in cameramen [13].

However, very few studies have included cameramen as research subjects, and no study has directly compared WRMDs in cameramen and other workers. The office workers were selected as a control group because of their lower physical demands compared to other occupations [16–18]. Therefore, we compared the incidence and severity of WRMDs among cameramen and office workers.

## Methods

### Subjects

Employees from four broadcasting companies in Korea were recruited to answer a questionnaire on musculoskeletal disorders. Studio cameramen (news, drama, and entertainment shows), video journalists, special cameramen (aerial or underwater filming), and cameramen who specialized in outdoor filming including those who filmed dramas, entertainment shows, sports games, and concerts were all classified as cameramen. Sedentary workers from the same broadcasting companies who spent most of their work time inside an office were classified as office workers. Employees who work indoors but can handle heavy objects are excluded. Of the 505 total subjects, 309 were office workers (42.72% women), and 196 were cameramen (1.53% women). Due to a significant discrepancy in the sex ratio, this study was limited to male subjects only. Subjects with current history of arthritis, gout, diabetes, and lupus were excluded. Subjects with less than one year of work experience were also excluded. A total of 293 subjects (129 cameramen and 164 office workers) were finally included in this study. A questionnaire survey regarding WRMDs and workplace ergonomics was conducted for one month starting in October 2016. We collected no personally identifiable information. Kangbuk Samsung Hospital Institutional Review Board approved our waiver of written informed consent (IRB No. KBSMC 2017–11–052-001).

### Musculoskeletal symptom and ergonomic evaluation

Musculoskeletal symptom questionnaires and the Manufacturing Operations Risk Factor (MORF)from the Korea Occupational Safety and Health Agency Guideline developed by DS Kim, JG Park, and GS Kim [19] were used to check WRMDs and ergonomic risks in the workplace. The questionnaires consist of five questions related to house labor, with possible answers including none, less than one hour, 1–2 h, 2–3 h, and over 3 h. Subjects were divided into two groups depending on whether the number of hours they spent on house labor was less than 1 h or greater than 1 h. Subjects who responded that they regularly exercise for more than 30 min at a time, twice per week, were assigned to the regular exercise group. They were also categorized according to whether their daily working hours were less than 9 h or greater than 9 h. Traumatic history was investigated for six different body parts, namely, neck, shoulders, arms/elbows, hands/wrists/fingers, waist, and legs/feet. Likewise, questions regarding work-related musculoskeletal pain and discomfort were answered for each of the six body parts to investigate if symptoms occurred bilaterally and to investigate symptom duration, symptom severity (mild, moderate, severe, and very severe), the incidence of symptoms in the last one year, and history of symptoms in the last one week. In accordance with The National Institute for Occupational Safety and Health standard, subjects who responded that they experienced moderate to very severe pain in any of the six body parts in the last one year, those whose WRMDs symptoms lasted more than one week, or those who experienced WRMDs symptoms more than once per month were assigned to the high-risk WRMDs group [20]. MORF was originally developed by the US Occupational Safety and Health Agency and University of Waterloo [21]. This assessment tool quickly assesses the ergonomic risks of the upper extremities, waist+lower extremities and manual material handling by investigating the posture, repeatability, pressure, vibration, and job control during working hours. The subjects’ responses to questions for the upper extremities and waist+lower extremities were weighted according to exposure time. Subjects who scored 10 or more points for the upper extremities and waist+lower extremities were considered to perform high-risk tasks for the corresponding body part.

### Statistical analysis

The general characteristics of cameramen and office workers were assessed separately. A chi-square test was used for the categorical variables, and an independent t-test was used for the continuous variables for comparison between the two groups. High-risk groups were identified for the upper extremities (neck, shoulders, arms, and hands/wrists/fingers) and the waist+lower extremities (waist and leg/foot) based on musculoskeletal symptoms reported by the subjects. High ergonomic-risk groups were identified for the upper extremities, and the waist+lower extremities depending on the high-risk tasks reported by the subjects. We used a multivariate logistic regression model to determine the odds ratios for musculoskeletal symptoms for each body part among cameramen compared with those among office workers with 95% confidence intervals (95% CIs). The model was adjusted for age in the first stage and then for tenure, regular exercise, daily working hours, house labor, traumatic history for each body part, and ergonomics in the second stage. The level of statistical significance was set at *p* < 0.05. The software program SPSS for Windows, version 24.0, (SPSS, Chicago, IL) was used for statistical analysis.

## Results

Cameramen had more tenure (year) and daily working hours compared to office workers. They also had more incidence of WRMD symptoms than office workers. No significant difference in all other variables was found between the two groups (Table 1).

**Table 1:** General characteristics of the study subjects

	Total	Cameramen	Office worker	*P* value
No. of participants (%)	293(100)	129(44.0)	164(56.0)	
Age, years (mean ± SD)	44.44 ± 7.48	45.23 ± 7.28	43.82 ± 7.61	0.110*
Tenure, years (mean ± SD)	16.35 ± 8.64	18.01 ± 7.98	15.04 ± 8.93	0.003*
Working hours/day (mean ± SD)	9.37 ± 2.39	10.42 ± 3.18	8.54 ± 0.87	< 0.001*
Marital status (%)				0.358**
Single	33(11.3)	17(13.2)	16(9.8)	
Married	260(88.7)	112(86.8)	148(90.2)	
Traumatic history (%)				0.479**
Yes	134(45.7)	56(43.4)	78(47.6)	
No	159(54.3)	73(56.6)	86(52.4)	
Regular Exercise (%)				0.308**
Yes	43(14.7)	22(17.1)	21(12.8)	
No	250(85.3)	107(82.9)	143(87.2)	
House labor/day (%)				0.284**
< 1 Hour	230(78.5)	105(81.4)	125(76.2)	
1 Hour ≤	63(21.5)	24(18.6)	39(23.8)	
WRMD symptom (%)				< 0.001**
Yes	148(50.5)	90(69.8)	58(35.4)	
No	145(49.5)	39(30.2)	106(64.6)	

*SD* standard deviation, *WRMD* work-related musculoskeletal disorder

*P value by independent t-test

**P value by Chi-square test

The subjects’ tasks were assessed using an ergonomics assessment tool (MORF). The high ergonomic-risk group for the upper extremities comprised a significantly higher proportion of cameramen (52.7%) than office workers (28.0%). The high ergonomic-risk group for the waist and the lower extremities also comprised a significantly higher proportion of cameramen (43.4%) than office workers (21.3%) (Table 2).

**Table 2:** Number and percent distributions of high-risk individuals in ergonomic evaluation according to body parts and occupation

	All	Cameramen	Office worker	*P* value*
Upper Extremities (%)				< 0.001
Normal	179(61.1)	61(47.3)	118(72.0)	
High-risk group	114(38.9)	68(52.7)	46(28.0)	
Waist+Lower Extremities (%)				< 0.001
Normal	202(68.9)	73(56.6)	129(78.7)	
High-risk group	91(31.1)	56(43.4)	35(21.3)	

*P value = by Chi-square test

Furthermore, the high-risk WRMD group for the upper extremities comprised a higher proportion of cameramen (32.6%) than office workers (11.0%). The high-risk WRMD group for the waist+lower extremities also comprised a higher proportion of cameramen (24.0%) than office workers (9.1%). The proportion of cameramen was significantly higher than that of office workers in the high-risk groups for all body parts except the hand/wrist/finger (Table 3).

**Table 3:** Number and percent distributions of high-risk individuals in work-related musculoskeletal disorder according to body parts and occupation

	All	Cameramen	Office worker	*P* value*
Upper Extremities (%)	60(20.5)	42(32.6)	18(11.0)	< 0.001
Neck	30(10.2)	21(16.3)	9(5.5)	0.002
Shoulder	45(15.4)	32(24.8)	13(7.9)	< 0.001
Arm	7(2.4)	7(5.4)	0(0.0)	0.003
Hand/finger/wrist	15(5.1)	9(7.0)	6(3.7)	0.201
Waist+Lower Extremities (%)	46(15.7)	31(24.0)	15(9.1)	0.001
Waist	40(13.7)	26(20.2)	14(8.5)	0.004
Leg/foot	15(5.1)	12(9.3)	3(1.8)	0.004

*P value = by Chi-square test

In the age-adjusted model, the OR for cameramen in the high-risk WRMD group relative to that for office workers was 4.03 (95% CI: 2.17–7.49) for the upper extremities and 3.18 (95% CI: 1.62–6.21) for the waist+lower extremities. The ORs by body parts were 3.38 (95% CI: 1.49–7.70), 3.85 (95% CI: 1.92–7.72), 2.77 (95% CI: 1.37–5.57), and 5.11 (95% CI: 1.40–18.66) for the neck, shoulders, waist, and the legs/feet, respectively, and were all statistically significant, except for the OR for hands/wrists/fingers. In the multivariate model, the ORs for cameramen in the high-risk WRMD group relative to that for office workers were 3.50 (95% CI: 1.76–6.96) and 2.43 (95% CI: 1.14–5.12) for the upper extremities and waist+lower extremities, respectively, and were all statistically significant. The ORs by body parts were 3.11 (95% CI: 1.28–7.57), 3.90 (95% CI: 1.79–8.47), and 4.23 (95% CI: 1.04–17.18) for the neck, shoulders, and legs/feet, respectively, and were all statistically significant, except for the ORs for the hands/wrists/fingers and the waist (Table 4).

**Table 4:** Age- and multivariate-adjusted odds ratio for high-risk WRMD group of cameramen using office worker as the reference

	Crude model	Age-adjusted model	Multivariate-adjusted model
Upper Extremities	3.92(2.12–7.23)	4.03(2.17–7.49)	3.50(1.76–6.96)
Neck	3.35(1.48–7.60)	3.38(1.49–7.70)	3.11(1.28–7.57)
Shoulder	3.83(1.92–7.66)	3.85(1.92–7.72)	3.90(1.79–8.47)
Arm	-^a^	-^a^	-^a^
Hand/Wrist/Finger	1.98(0.68–5.70)	1.97(0.68–5.71)	1.59(0.47–5.42)
Waist+Lower Extremities	3.14(1.61–6.12)	3.18(1.62–6.21)	2.43(1.14–5.12)
Waist	2.71(1.35–5.43)	2.77(1.37–5.57)	2.05(0.92–4.59)
Leg/Foot	5.50(1.52–19.94)	5.11(1.40–18.66)	4.23(1.04–17.18)

Multivariate model adjusted for age, tenure, regular exercise, daily working hours, house labor, traumatic history, ergonomic risk

Values are odds ratio (95% confidence interval)

*WRMD* work-related musculoskeletal disorder

^a^No office worker in high-risk work-related musculoskeletal disorder group

## Discussion

Previous studies have measured the level of physiological stress experienced by cameramen in terms of heart rate and oxygen consumption and classified cameramen’s tasks as physiologically heavy work [15], analyzed occupational suprascapular nerve entrapment among cameramen [22], and identified ergonomic factors and physical burden as risk factors of WRMDs in cameramen, which were found to affect the shoulders most commonly [14]. However, these studies limited their research subjects to cameramen only. In the present study, we compared WRMDs between cameramen and office workers and found that the risk of WRMDs was higher for cameramen. When break time is reduced due to long working hours, musculoskeletal fatigue can occur and induce WRMDs [23]. Chronic musculoskeletal fatigue can lead to accumulated stress on muscles and tendons and subsequently, reduced blood flow in the corresponding areas as work experience and age increase. Eventually, they cause pain and poor function by inducing localized muscle fatigue [24].

Ergonomic stress in the work environment is a risk factor for WRMDs [13]. The biomechanical risks of WRMDs include repetitious and forceful work, awkward or sustained posture, and mechanical pressure [13]. The cameramen’s jobs are associated with these risk factors. Cameramen can also be exposed to ergonomic risks depending on the type of camera and filming methods they use. Standard studio cameras used in studios have wheels attached to them for mobility. Although these cameras are usually used in the erect state, cameramen are forced to repeatedly use pulling and pushing forces to change the filming angle and distance. While filming, they must twist their bodies and tilt their head up to look at the viewfinder for monitoring. There are three main methods of outdoor filming: filming using a tripod, filming by directly holding the camera, and filming using a jimmy jib camera. When filming with a camera fixed in a tripod (Electric News Gathering Camera, Electronic Field Production camera, Electric cinematography), cameramen must squat or bend their waist to adjust the height according to the position of the subject, and thus cannot film in a comfortable posture. Their bodies twist, and their necks bend downward to look at the viewfinder. When filming by directly holding a camera (Electric News Gathering Camera), cameramen must carry the weight of the camera, which is over 10 kg. They usually place the camera on their right shoulder for a long period of time without moving and must use different postures such as lifting the camera above the shoulder or lowering it to below the waist or knee level depending on the situation. GK Karatas and F Gogus [22] proposed that these risk factors induce suprascapular nerve entrapment among cameramen. Meanwhile, a jimmy jib camera in the shape of a long lever is used to get an overall shot from high places. A weight is added to one end of the lever and a camera at the other end to adjust the camera height. It is the cameramen’s job to lift the weight above the head or lower it below the knees. Outdoor filming requires continuous angle adjustment, focus, and light exposure before the actual shooting begins. Therefore, they should be considered as working hours for the cameramen other than moving hours and resting hours. And the cameramen’s filming posture can vary for each scene, each angle.

Office workers can also develop musculoskeletal symptoms of the waist, neck, shoulders, and hands/wrists/fingers due to long hours of using computers [25–27]. However, as can be deduced from the characteristics of cameramen’s tasks described earlier, cameramen appear to be at higher ergonomic risk than office workers and thus showed higher ORs for WRMDs of the neck, shoulders, and legs/feet in this study. The reason why the risk of WRMDs was high for the neck may be that cameramen look downward or upward while filming. The shoulders were susceptible to WRMDS possibly because cameramen place the cameras on their shoulders for long periods. Meanwhile, the reason why the risk of WRMDs was high for the legs/feet may be that cameramen often have to stand or squat, whereas office workers spend most of their working hours sitting.

The present study had several limitations. First, musculoskeletal symptoms and ergonomic risk factors were assessed based on self-reported answers. Subjects may have overreported their symptoms and the ergonomic aspects of their occupations. However, because it was difficult to diagnose the subjects with a specific musculoskeletal disorder and cameramen performed a wide range of tasks, there were limits to assessing their symptoms using the Rapid User Limb Assessment [28] or the Rapid Entire Body Assessment [29]. As such, it was difficult to use objective markers. In the following study, it would be helpful to use an objective evaluation tool, such as the video-based observation method [30]. Because different kinds of cameras used for different tasks were clustered to one group and assessed together in this group, future research must divide them further according to their specific tasks. Lastly, all female subjects were excluded, and only male subjects were studied. However, had women, who have higher sensitivity to musculoskeletal symptoms than men [13], been included, the risk of musculoskeletal symptoms among cameramen would have increased. Thus, it will not be difficult to obtain results even when subjects are limited to men only.

Due to media advancements and increased value of media contents, the demand for cameramen has increased. However, research on cameramen, who work in unfavorable work environments that impose long working hours and short break time, who perform ergonomically dangerous tasks that involve making awkward postures and withstanding heavy weights, and who are exposed to various risk factors for WMRD because of the nature of their work, is lacking. In this study, the WRMD symptoms and ergonomic factors were assessed to not only demonstrate that the risk of WRMDs is higher among cameramen than among office workers, but also to show that the tasks performed by cameramen pose high ergonomic risks. No study has directly compared musculoskeletal symptoms between cameramen and other occupations. Confounding effects were minimized by comparing the cameramen with office workers from the same broadcasting companies.

## Conclusion

Cameramen were at higher risk for WRMDs than office workers. The risk of WRMDs of the shoulders, neck, and legs/feet was higher than that of other body parts. The working hours of cameramen should be reduced, and the workplace environments with high ergonomic risks should be improved. Furthermore, prevention and follow-up management of WRMDs among high-risk occupations such as cameramen are important. Management of WRMDs affecting the neck, shoulders, and legs/feet, which are highly susceptible to WRMDs, may be effective.

## Abbreviations

95% CI: 95% Confidence interval
KOSHA: Korea Occupational Safety and Health Agency
MORF: Manufacturing Operations Risk Factor
OR: Odds ratio
SD: Standard deviation
WRMDs: Work-related musculoskeletal disorder
